# Natural Anti-Estrogen Receptor Alpha Antibodies Able to Induce Estrogenic Responses in Breast Cancer Cells: Hypotheses Concerning Their Mechanisms of Action and Emergence †

**DOI:** 10.3390/ijms19020411

**Published:** 2018-01-30

**Authors:** Guy Leclercq

**Affiliations:** Laboratoire de Cancérologie Mammaire, Institut J. Bordet, Centre des Tumeurs de l’Université Libre de Bruxelles, 1, rue Héger-Bordet, 1000 Brussels, Belgium; guy.leclercq@ulb.ac.be

**Keywords:** estrogen receptor α, natural antibodies, estrogenic responses, mechanism of action, auto-immune diseases

## Abstract

The detection of human anti-estrogen receptor α antibodies (ERαABs) inducing estrogenic responses in MCF-7 mammary tumor cells suggests their implication in breast cancer emergence and/or evolution. A recent report revealing a correlation between the titer of such antibodies in sera from patients suffering from this disease and the percentage of proliferative cells in samples taken from their tumors supports this concept. Complementary evidence of the ability of ERαABs to interact with an epitope localized within the estradiol-binding core of ERα also argues in its favor. This epitope is indeed inserted in a regulatory platform implicated in ERα-initiated signal transduction pathways and transcriptions. According to some experimental observations, two auto-immune reactions may already be advocated to explain the emergence of ERαABs: one involving probably the idiotypic network to produce antibodies acting as estrogenic secretions and the other based on antibodies able to abrogate the action of a natural ERα inhibitor or to prevent the competitive inhibitory potency of released receptor degradation products able to entrap circulating estrogens and co-activators. All of this information, the aspect of which is mainly fundamental, may open new ways in the current tendency to combine immunological and endocrine approaches for the management of breast cancer.

## 1. Introduction

Among modulators of steroid hormone receptors, natural anti-estrogen receptor antibodies (ERABs) are of peculiar interest in view of their implication in the emergence and/or evolution of autoimmune diseases and cancers [1]. The present paper focuses on the potential biological relevance of these antibodies in the context of the hormone-dependence of breast cancer, a topic on which I have been working for more than four decades.

The recent finding by the group of Pierdominici and Ortona of a correlation between the titer of ERABs raised against the alpha form of the receptor (ERαABs) in sera from a series of women with breast cancer and the percentage of Ki67-positive cells (a known marker of proliferation) in samples taken from their tumors [2] offered to me an opportunity to discuss here the possible implication of these antibodies in the development of breast cancers. In fact, this concept had already been proposed in the late 1980s by my colleague Borkowski, who detected a sub-population of IgGs able to interact with the estradiol (E_2_) binding site of ERα in sera from healthy women [3,4]. This work, in which I collaborated, revealed moreover the ability of these IgGs to induce estrogenic (or estrogenic-like) responses in ERα-positive MCF-7 breast cancer cells, suggesting that they act on these cells as the hormone [4]. Further studies revealed that this view was only partly true: the major estrogenic activity of the IgGs seemed to derive from the neutralization of ERα-related peptides able to inhibit its activation [5]. Skepticism concerning the biological significance of these various observations, as well as their potential insertion in therapeutic programs forced us to stop our investigations. We hope that the recent investigations of Pierdominici and Ortona, which also concern the prominent role of estrogens in autoimmune diseases [6], may encourage the scientific community to assess again questions relevant to the suspected role of such natural anti-ERα antibodies in breast cancer.

The present paper devoted to this hope mainly concerns the mechanism(s) by which ERαABs may operate; processes implicated in their emergence will be also evoked. Available data being quite tenuous, my proposals are largely speculative. Nevertheless, I anticipate that they may open avenues for new experimentations not necessarily restricted to ERα, since the existence of natural antibodies raised against other steroids hormone receptors has been reported, as will be recalled briefly in the next section.

## 2. Natural Antibodies against Steroid Hormone Receptors, the Existence of Which Had Been Reported about Three Decades Ago

To my knowledge, the first evocation of such antibodies must be attributed to the group of O’Malley that reported in 1981 the existence of “spontaneous” antibodies raised against the progesterone receptor in two thirds of sheep sera [7]. Surprisingly, these authors limited their investigation to the assessment of the binding properties of these antibodies for the α and β isoforms of this receptor without raising any questions relevant to their biological role. This topic was addressed in the following year by Liao and Witte who reported a high titer of anti-androgen receptors in patients with prostate disease, when compared with normal subjects [8]. These authors logically proposed some relevance to this detection in terms of disease management. The discovery of the existence of anti-ERα may be ascribed to Borkowski [3], as well as to Muddaris and Peck Jr. [9], who detected them at the same time. While Borkowski focused his studies on the biological function of these antibodies, Muddaris and Peck reported striking sex and age-related differences in the level of the latter: young females displayed a higher titer than corresponding males. This level also declined in middle age, before increasing in old age, in contrast to males in which it continuously decreased. Although these various observations were quite provocative, they failed to generate a significant interest for about two decades, as previously mentioned.

## 3. Major Properties of ERαABs

### 3.1. Ability to Induce Estrogenic (or Estrogenic-Like) Responses

As reported below, ERαABs act as ERα agonists through both non-genomic and genomic procedures, which operate sequentially, the non-genomic preceding largely the genomic procedures [10,11]. This suspected co-operative mechanism [11,12,13,14], detected with MCF-7 breast cancer cells, seems to be initiated at the plasma membrane (Section 3.2).

#### 3.1.1. Signal Transduction Activation and Subsequent Cell Proliferation Enhancement

Highly purified ERαABs almost immediately activate the phosphorylation of ERK (Extracellular regulated kinase) in MCF-7 cells without producing any similar effect on Akt (Protein kinase B) [2]. The maximal effect of the antibodies occurs after 5 min and subsequently declines, returning to the original level after 30 min. As expected, a significant increase in proliferation is recorded after one day of treatment.

#### 3.1.2. Transcriptions and Related ERα Level Changes

Over-night exposure of MCF-7 cells to highly purified ERαABs (IgGs) enhances their level of progesterone receptors in a dose-dependent manner, as observed with E_2_ used as the control; this increase is progressively inhibited by pure antiestrogens [4,5]. The same behavior is recorded for cathepsin D secretion. A loss of the capacity of the cells to specifically incorporate [^3^H]E_2_ (ERα whole cell assay) occurs in parallel, which may be ascribed to a decrease of the ERα level, detected by Western blotting. IgGs also partially abrogate the capacity of the cells to incorporate [^3^H]E_2_ in the presence of an analog of hydroxy-tamoxifen, which stabilizes the receptor within the nucleus [15], as does E_2_.

### 3.2. Selective Ability to Associate with the E_2_-Binding Site of the Native Form of ERα Localized at the Plasma Membrane

**Figure 1:**
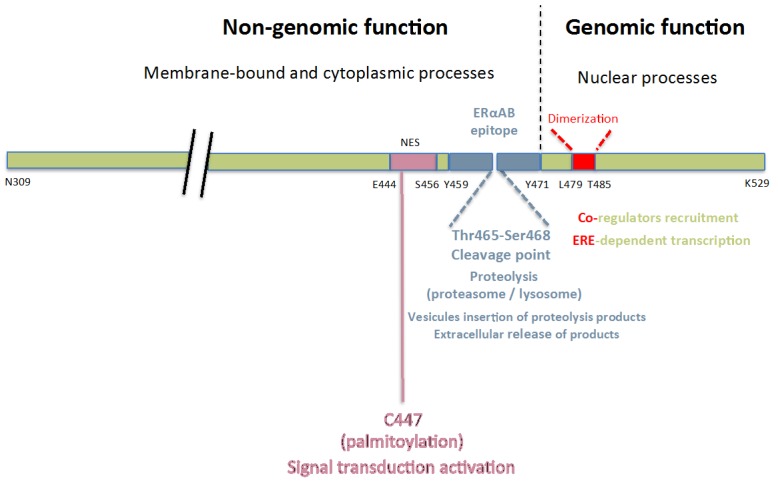
Schematic representation of the regulatory platform of the E_2_-binding core of ERα (N309-K529), postulated to mainly contribute to the onset of non-genomic and genomic responses induced by E_2_ and ERαABs. The ERαABs’ epitope (Y459-E471) occupies a central, pivotal position localized between two motifs, each of them being implicated in one of these two types of responses (non-genomic, E444-S456; genomic, L479-T485). Functions of these three amino-acids sequences, as well as biological consequences resulting from E_2_/ERαABs binding and consecutive activation of related inter-relationships between motifs of the platform are defined below (for details, see Section 3.3).

When submitted to low-salt sucrose gradient sedimentations, ERα from cytosolic extracts is known to migrate within two distinct oligomeric structures, i.e., of 4 and 8S (note that these velocities may slightly differ according to the nature of the tissues from which ERα is extracted, the experimental conditions, as well as the choice of the sedimentation markers used for their assessment) [16,17]. The 4S entity contains proteolytic products of the receptor, while the latter is maintained within the 8S entity in its native form (67 kDa) by a protective action of chaperones with which it associates. Interactions between highly purified ERαABs (IgGs) and ERα occur in the region of its E_2_-binding site since an increase of sedimentation velocity of the 8S oligomer is detected when the [^3^H]E_2_ labeling of the receptor is performed after sedimentation on the fractions collected from the gradient. With pre-labeled cytosols, this sedimentation shift is replaced by a partial displacement of bound [^3^H]E_2_ by the IgGs [3]. Complementary experiments including an assessment of the binding parameters of [^3^H]E_2_ to ERα, in the absence and the presence of increasing amounts of these IgGs, respectively, confirmed the implication of the E_2_ binding site of the receptor in this complex. Accordingly, these IgGs behaved as competitive inhibitors (increase of *K*_d_ values) [3], a finding in agreement with the recent identification of an epitope able to recognize ERαABs (Y^459^TFLSSTLKSLEE^471^; Figure 1) within the E_2_-binding core of ERα (Asn309-Lys529; MW: 26 kDa [18]) [2].

Interestingly, this Tyr459-Glu471 epitope contains a small motif (Thr465-Ser468), which is cleaved under proteolytic attack without any loss of E_2_ binding ability [18], a property resulting from a cutting of the estrogen-binding core of ERα in two distinct entities (7 and 17 kDa) that stick together through hydrophobic contacts [19]. According to our sedimentation data, such a complex would logically be sufficient for ERαABs recruitment by the “pseudo” native ERα when it is stabilized in a peculiar oligomeric quaternary structure. Hence, one may understand that the known dissociation of such a structure at the time of ERα activation under the action of an appropriate modulator affects the topology of the ERα-binding core, giving rise therefore to a loss of its recruitment potency for ERαABs, a property that manifestly does not hold for E_2_ and most probably other conventional estrogenic ligands [20,21,22,23].

This suspected binding selectivity, as well as the large size of ERαABs may explain their association in living cells with the plasma membrane-bound receptor form (mERα) [2], principally localized within caveolae [10,11]. This peculiar localization, which results, at least in part, from the palmitoylation of the native (newly-synthetized) receptor [13], appears especially appropriate for this association contributing to rapid, non-genomic, responses (in the present context, ERK phosphorylation; Section 3.1.1). It does not indeed imply any navigation of ERαABs across the plasma membrane to reach oligomeric complexes in which they would moreover not easily internalize to interact with the native and non-markedly altered receptor forms.

### 3.3. Potent Regulatory Functions of the ERαAB-Binding Epitope

The sensitivity to proteolytic attacks of the Thr464-Ser468 amino acid sequence of the ERαAB-binding epitope of ERα suggests its inclusion within a surface-exposed region, a property usually recorded with “regulatory platforms” subjected to recruitment and exchange of co-regulators [24]. The identification within this epitope of two functional motifs localized respectively on the left and right sides of the ERαAB-binding epitope supports such a view.

The left-side sequence (E^444^FV**C**LKSIILLNS^456^; Figure 1) corresponds indeed to an identified nuclear exclusion signal that contributes to the return of the activated ERα within the cytoplasm, where it is subjected to proteasomal degradation [24,25,26,27]. This step is key for the pursuit of previously initiated transcriptional processes. Hence, this motif would play a role in ERα intracellular trafficking as well as in its resulting turnover rate and related biological activity [11,13,24]. The presence within this motif of Cys-447, the palmitoylation of which favors the anchorage of the receptor with the plasma membrane, validates this proposal [28]. In contrast, the right-side motif (L^479^DKTITDT^485^) seems mainly to contribute to (Estrogen response element) ERE-dependent transcription since it corresponds to one of the three amino-acids sequences of the ERα homo-dimerization interface required for such transcription [29].

Hence, the pivotal position of the Thr465-Ser468 sequence within the E_2_-binding core of ERα (which contains the ERαABs binding epitope) confers to this sequence a primordial role in the onset of quasi-immediate non-genomic responses, as well as subsequent genomic responses. Such a dual capacity of action is reminiscent of a model proposed to explain how a ligand of the so-called nuclear receptor family may activate rapid signal transduction pathways issued from the cellular membrane, as well as genes’ expression, either individually or sequentially [30,31].

According to this model, all ligands’ binding sites of the nuclear receptor family are composed of two adjacent cavities in which potent agonists and antagonists may penetrate [30,31]; for ERα and β, see [32]. One of these cavities corresponds to a channel conducting to the other cavity in which selected ligands may be engulfed; the capacity of the ligands to open a protective barrier localized at the entrance of this second cavity might regulate this selection. Molecular interactions between receptors, chaperones and co-regulators are also implicated in this access-regulatory process. The entrance channel, in which access is less restrictive, is directly implicated in quasi-immediate activation of signal transduction pathways, while the cavity in which ligands are engulfed corresponds to the pocket contributing to receptor-mediated transcription, the topology of which has been established by X-ray diffraction crystallography. Cellular localization of the receptor is logically a complementary factor involved in this dual regulation.

Logically, the rapid ERαAB-induced ERK phosphorylation implicated in the enhancement of MCF-7 cells’ proliferation (Section 3.1.1) may derive from a relatively low specific interaction of these antibodies with the entrance cavity, the structure of which may be related to the left-side motif implicated in non-genomic responses. Such a hypothesis may also hold to some extent for subsequent indirect induction of ERE-dependent transcription, since this left-side motif seems to play a role in the intracellular trafficking of the receptor, which regulates such transcription. ERαAB-mediated enhancement of the progesterone receptor level may obviously not result from an engulfment of these antibodies within the putative adjacent cavity implicated in gene expression. Access to this adjacent cavity being under the control of a barrier, one may propose that interactions between ERαABs and specific residues of the entrance cavity in which they may penetrate would suppress the repressive function of the barrier, favoring thereby ERα-mediated transcriptions. Receptor-related binding motifs of the plasma membrane may contribute to this property.

## 4. ERα-Related Sites Potentially Able to Contribute to the Mechanism of Action of ERαABs

Several sites identified on the plasma membrane may legitimately be proposed as potential alternative targets for ERαAB-induced responses, some of them acting cooperatively with mERs [33]. Some of these sites are devoid of any E_2_-binding ability (i.e., HER2, EGFR), while others attract the hormone as demonstrated with synthetized E_2_-conjugates unable to penetrate the cells [34]. Among such E_2_-binding targets, two splice receptor variants (ERα36 and ERα46; see [35,36] and the references therein), as well as a G protein-coupled receptor (GPR30 [37,38,39]) have been especially well studied. The capacity of GPR30 to interact with calmodulin, as well as with the calmodulin-binding site of ERα, implicating its dimerization for the enhancement of ERE-dependent transcription [40,41,42,43], would confer to this peculiar receptor a potent role in ERαAB-induced genomic functions. In fact, the capacity of GPR30 to move between the plasma membrane, the endoplasmic reticulum and the nucleus advocates in favor of its contribution to other ERα-mediated processes under the control of the antibodies [37,44]: GPR30 appears indeed to be an actor involved in the intracellular trafficking of the receptor governing its various biological functions.

Of course, the implication of such receptor-related sites in the onset of ERαAB-induced responses needs to be validated or rejected. Measurement of markers (Ca^2+^ fluxes or secondary messengers such as c-AMP or IP3) may be helpful in this regard, especially for the evaluation of complementary ERα-independent processes [33]. In this context, specific antagonists with a special emphasis on compounds abrogating the action of HER2, EGFR or GPR30 need also to be tested. This approach being at the present time quite marginal [2,5], one may consider that any use of such antagonists in the clinical perspective is out of scope, even if humanized versions of monoclonal antibodies raised against HER2 (trastuzumab, pertuzumab) seem appropriate for a first-line experimental assessment [45,46]. Induction by such drugs of a decrease of efficiency of signal transductions initiated by the putative action of ERαABs at the level of HER2 might alter growth of breast cancer cells, which in connection with the known antibody-dependent cellular cytotoxicity (ADCC) of these compounds related to their ability to recruit and activate natural killer cells (NK) would generate a major curative effect, even in the absence of ERα. Note in this context that pertuzumab abrogates the hetero-dimerization of HER2 with other members of the HER family, while trastuzumab mainly affects its homo-dimerization. Since such dimerizations are implicated in the activation of signal transductions enhancing cell growth, one may consider that pertuzumab might be more efficient for blocking a putative ERαABs association with membrane ERα-related receptors, promoting proliferation.

Finally, it should be stressed that the estrogen activity of ERαABs should not necessarily be derived from a direct interaction with the plasma membrane-bound E_2_-binding site. This assumption results from experiments conducted with anti-E_2_~BSA antibodies and highly purified ERαBAs (IgGs), which displayed an estrogenic activity [5]. Anti-E_2_~BSA antibodies sharing most likely some structural similarities with the hormone binding site of ERα, the authors of this observation concluded that these two classes of antibodies may act as “soluble ERα forms” present in the blood to liberate, by a competitive process, the receptor from the repressive action exerted by a peptide inhibitor looking structurally like E_2_~BSA. Hence, the estrogenic activity of a subpopulation of ERαABs may result, at least in part, from the ability to abrogate the effect of ERα co-repressors. If confirmed, this concept would logically also hold for other possible ERαABs targets, as described below.

**Figure 2:**
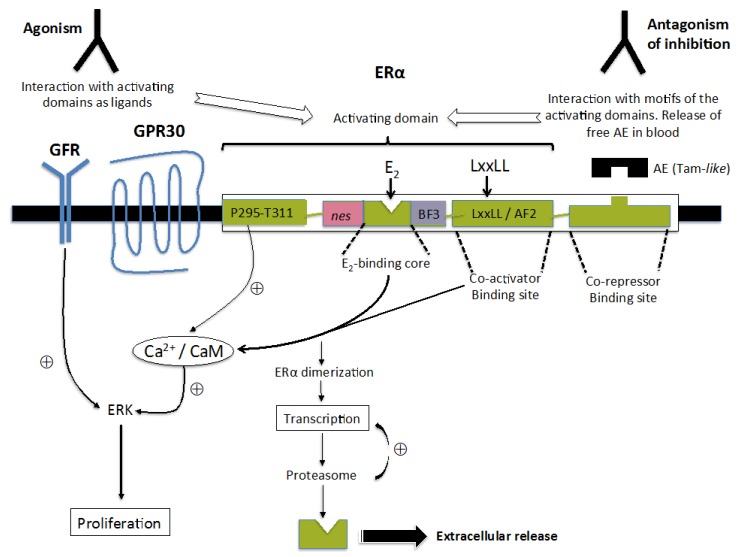
Schematic representation of ERαABs-induced mechanisms initiated at the plasma membrane to promote enhanced proliferation and ERE-dependent transcription. Reported ERαAB activities (agonism, antagonism of inhibition) were integrated in a classical model explaining co-operation between binding sites for growth factors and steroid hormones in the onset of non-genomic and genomic responses [11,12,14,27,33,37,38]. Note the pivotal role of the Ca^2+^/calmodulin complex in the inter-relationships between GPR30 and recruitment sites of the receptor for ERαABs and adjacent co-activators. AE: antiestrogen; Tam-like: Tamoxifen-like.

All hypotheses evoked in Section 3 and Section 4 to explain the mechanisms by which ERαABs may generate estrogenic responses are schematically summarized in Figure 2.

## 5. Mechanisms Implicated in the Emergence of ERαABs

The present section will solely refer to the emergence of ERαABs for which ERα binding properties have been overviewed. For any topics concerning E_2_-related changes in immune functions or auto-immunity, I invite the reader to consult [1,6], which are extensive in this regard.

**Figure 3:**
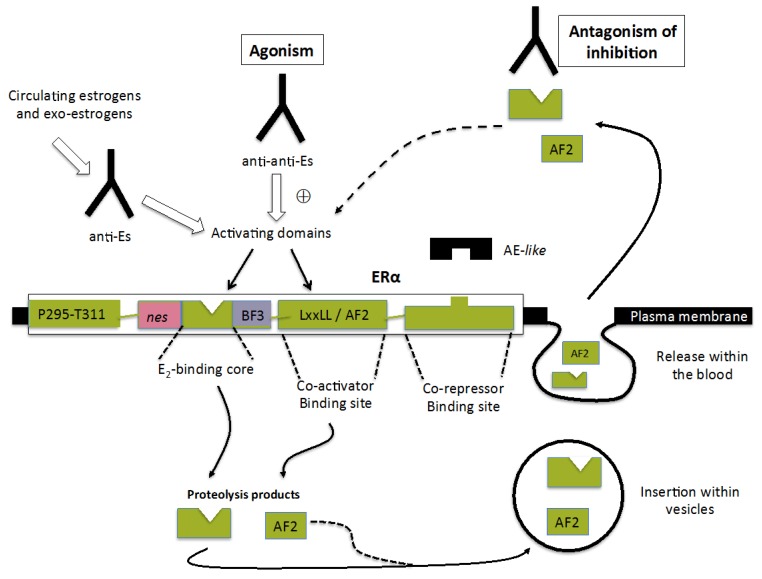
Schematic representation of suspected mechanisms able to contribute to ERαABs’ emergence. Agonists: antibodies able to mimic the action of circulating estrogens (natural, synthetic and xenoestrogens). Anti-antagonists: antibodies against a natural extra-cellular repressor recognizing a specific inhibitory site of ERα or preventing the access of activating modulators to the receptor by a competitive binding process. ERα degradation products including binding sites for estrogens or LXXLL motifs of co-activators [24], released within the blood, may generate this last class of antibodies. Es: Estrogens, BF3: Binding function 3, AF2: Activation function 2.

For me, insufficient experimental data have been reported to propose mechanisms giving rise to the production of ERαABs. A priori, two auto-immune reactions involving eventually a contribution of the idiotypic network may theoretically be advocated, as suggested in the previous sections: one giving rise to antibodies acting as endogenous estrogenic secretions or any expositions to environmental estrogens, the other to antibodies abrogating the repressive action of a natural antagonist. In this context, one may wonder about the participation of recently identified circulating ER (α and β) forms in human sera in the emergence of ERABs (see Section 5.3). The next sections will analyze the relevance of these possibilities, schematically presented in Figure 3.

### 5.1. Anti-Idiotypic Antibodies Acting as Physiological Estrogens

Similarities between E_2_ and ERαABs, in terms of interactions with the native ERα form, reflect most probably structural identities between the hormone and the active site of natural anti-idiotypic antibodies raised against anti-E_2_ IgGs. Since circulating E_2_ is mainly conjugated to serum proteins, one may postulate that such a cross-reaction may also hold for such conjugates, especially anti-E_2_~BSA IgGs, the level of which would largely dominate. Since such a concept is not restrictive to this hormone, it should be extended to all other physiological estrogens, as well as so-called “xenoestrogens” (natural phytoestrogens and synthetic “endocrine disrupting chemicals” [22,23]) able to interact with the ligand-binding site of ERα to induce estrogenic (or estrogenic-like) responses. The implication of these molecules in autoimmune response has been, indeed, evoked [47].

The production in the early 1990s of a monoclonal antibody (clone 1D5) directed against the binding site of an anti-E_2_ monoclonal antibody lends credence to this concept: 1D5 was found to interact with the hormone-binding domain of the receptor to mimic some estrogenic actions (creatine kinase induction, rapid Ca^2+^ flux enhancement), both in vivo and in vitro [48,49]. These experimental data were proposed to be mainly dependent on an interaction with a membrane-bound form of ERα. Nevertheless, they were also postulated to result to some extent from an intra-cellular penetration of 1D5. A lack of knowledge concerning the relationships between the plasma membrane and intra-cellular ERα forms at the time of such studies may explain this statement, which may appear now quite obsolete, even if this could not be excluded.

### 5.2. Antibodies with Anti-Repressive Activities

In theory, all receptor-mediated agonistic activities may result from an ability to abrogate the antagonism of a modulator acting at the level of the ligand-binding site or an adjacent site implicated in the recruitment of co-repressors. Such a view has been proposed to explain, at least in part, the estrogenic activity of ERαABs (Section 4, last paragraph). While a potency to liberate the E_2_-binding site of the plasma-bound receptor from the antagonism of a specific inhibitor has been solely addressed [5], it seems that other sites of the hormone-binding domain involved in the recruitment of co-activators (LXXLL/AF2, BF3, etc. [24,50]) must also be taken into account. However, the presence of such sites in the whole family of steroid hormone receptors would largely limit the specificity of action of antibodies raised against them, giving rise to inappropriate adverse effects. Hence, their importance seems quite dubious.

In this context, a possible interaction of ERαABs with an identified ERα-binding site implicated in the recruitment of tamoxifen and other mixed antagonists/agonists [51,52] may also be advocated. Experiments revealed that that treatment of cytosolic ERα preparations with tamoxifen enhances the immuno-reactivity of this site for a monoclonal antibody (H222) raised against an epitope of the receptor ligand-binding domain [53], revealing that this compound may expose an occult antigenic determinant accessible to a subpopulation of ERαABs. Whatever could be the finality of such an interaction with a site contributing to the activity of tamoxifen, either agonist or antagonist, one may consider that it may modulate the SERM character of this compound.

### 5.3. Implication of ERα or ERα Fragments Released within the Blood in the Onset of ERαABs

Could ERα and β recently detected within human sera [54] be implicated in the emergence of ERαABs against these two receptors? This important question has some justification in the finding that the latter display anti-inflammatory properties, the net action of which depends on their relative proportions (β > α) and localization; ERα is moreover associated with auto-immune processes [55].

These circulating ERα and β forms (detected in patients with Crohn’s disease with a commercial ELISA) most probably correspond to various receptor fragments issued from their intracellular proteasomal and lysosomal degradation, released within the blood as small vesicles (exosomes) implicated in immune responses or processed for MHC (major histocompatibility complex) presentation after autophagy [56,57]. Hence, one may logically assume that ERα fragments may be implicated in the emergence of ERαABs with a repressive activity, some of them abrogating the effect of natural inhibitors present in the blood, others abrogating the potent competitive inhibitory potency of ERα degradation products able to recruit circulating activators (mainly E_2_, co-activators), liberating thereby these agents for the accomplishment of their function.

The detection in media from E_2_-stimulated cells of a 44-amino-acid peptide including a repressive motif of ERα (Pro295-Thr311) [58], able to interact with the Pro365-Asp369 type II β turn element of its BF3 motif that regulates the dimerization of the receptor ([59], and see Section 6), may appear as a stone in the edification of this concept. A synthetic peptide corresponding to the Pro295-Thr311 motif (ERα17p) induces indeed estrogenic responses, as well as some receptor-independent actions in various breast cancer cell lines [60], the lack of specificity of these actions resulting most probably from distinct interactions with the type II β turn/BF3 motifs of the various steroid hormone receptors expressed in these cells. Antibodies raised against the P295-T311 sequence (Gentaur: 04-rb-ERα17p) would logically generate a similar absence of specificity of action in contrast to antibodies raised against the E2-binding core of the receptor (Section 5.2, first paragraph). Such a lack of specificity would not be necessarily detrimental for therapeutic purposes, especially in the case of antiestrogen resistance, as proposed for antagonists aimed to antagonize the recruitment of co-activators [24,61].

## 6. Conclusions and Perspectives

**Figure 4:**
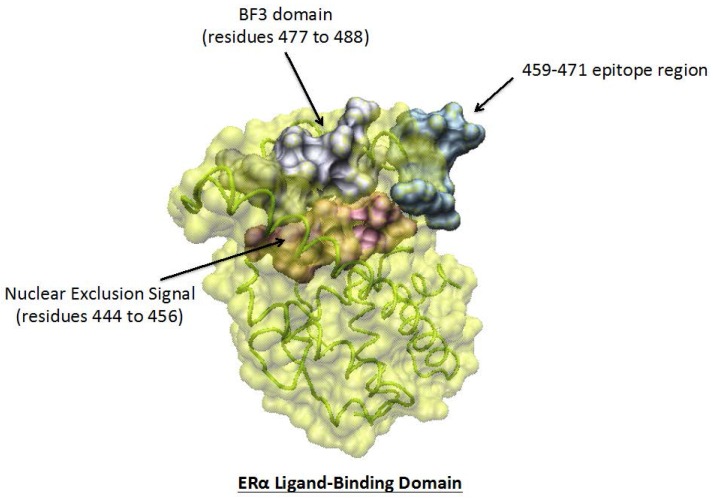
Surface structure of the BF3- (in grey) and ERαAB epitope/E_2_ (in blue)-binding domains of the human estrogen receptor α (ERα in yellow, Connolly surface). The BF3 domain is composed of two regions, i.e., the 365–369 type II β-turn region and the 477–488 helix 10 (H10) region, the latter overlapping the 479–485 sequence implicated in the dimerization of the receptor. The ERαABs epitope is in close contact with this BF3 domain, as well as the 444–456 nuclear exclusion site (nes, for nuclear exclusion signal, in pink). Interaction between the 301–311 region with the 365–269 type II β turn seems to repress the dimerization potency of the 477–488 helix. Transparency allows the visualization of the helices (in green) that comprise the receptor.

Structural studies of the estrogenic core of ERα, reported here, reveal that the ERαABs epitope localizes at a place of prominent importance for the successive onset of non-genomic and genomic responses. The finding that this epitope is adjacent to regulatory motifs governing these responses argues in favor of such a statement. Complementary inclusion of these data into a model established from X-ray crystallographic investigations relevant to the activated intracellular ERα form indicated that the Leu479-Thr485 motif, which contributes to the dimerization of the receptor, corresponds to a part of its BF3 motif implicated in ERE-dependent transcription [61] (Figure 4; analysis performed by my colleague Yves Jacquot, Sorbonne Universités, Université Pierre et Marie Curie, Ecole Normale Supérieure, Paris, France). This information strongly suggests that ligand-induced conformational changes relevant to the intracellular receptor may also hold for its plasma membrane-bound form, justifying the dimerization ability of the latter. Hence, the biochemical assessment of the interactions between ligands aimed at targeting the “insoluble” ERα entrapped within the plasma membrane and the conventional “soluble” cytoplasmic and nuclear receptor forms would be a valuable approach to the decryption of the mechanism by which ERα operates. Hence, interest in ERαABs would not be restricted to physio/pathological purposes.

In this regard, experimental data critically reviewed here leave no doubt about the importance of ERαABs in breast cancer emergence and/or evolution, even if these biological aspects have only been marginally addressed at the present time [2,5]. The capacity of ERαABs to stimulate MCF-7 cell growth suggests some potential implication in the resistance to endocrine treatments. Such a topic needs to be rapidly assessed with tamoxifen-resistant cell lines. On the other hand, since the mammary gland is under the control of both ERα and β, which respectively promote or repress its neoplasia [62], the search for natural antibodies raised against ERβ seems of major interest. Such a task may open new pathways in the current tendency to combine immunological and endocrine approaches in the management of cancer. The present review being mainly devoted to fundamental aspects of ERαABs, I encourage immunologists and endocrinologists to extend my work to reported clinical observations, especially those that, by ignorance, I failed to refer. Such an issue will be extremely helpful to confirm or reject a tendency to see a strong autoimmune ER function in breast cancer, which, in the affirmative, would be taken into account in the design of future therapeutic programs.

## Abbreviations

Akt	Protein kinase B
AF2	Activation function 2
BF3	Binding function 3
ERK	Extracellular regulated kinase
EGFR	Epidermal growth factor receptor
ER	Estrogen receptor
ERAB	Estrogen receptor antibody
ERE	Estrogen response element
HER 2	Human epidermal growth factor 2
IP3	Inositol triphosphate
